# Supplementary material for: Are carbon emissions trading and green financial instruments synergistic? -Comprehensive quantitative research based on content analysis
Source: PLoS One. 2024 Mar 7;19(3):e0298601. doi: 10.1371/journal.pone.0298601 (PMC10919601; doi:10.1371/journal.pone.0298601)
Supplement: S1 Data — (ZIP) [file pone.0298601.s001.zip › Data/地级市Z_F工作报告.zip!/╜¡╬≈/╨┬╙α╩╨/└┤╫╘╣1⁄2╓┌║┼í╛┬φ┐╦╩2╛▌═°í┐_2014.docx]

各位代表：

　　现在，我代表市人民政府向大会报告政府工作，请予审议，并请各位市政协委员和其他列席会议的同志提出意见。

　　一、2013年工作回顾

　　2013年，面对复杂严峻的国内外形势，全市上下在省委、省政府和市委的正确领导下，以党的十八大精神为指引，深入贯彻落实科学发展观，牢牢把握稳中求进工作总基调，群策群力、开拓创新，有力地推动了全市经济社会平稳健康发展。

　　这一年，是攻坚克难奋发有为的一年。一年来，我们沉着应对各种挑战，积极化解各种风险，确保了经济发展阶梯提速、稳中有升。预计（下同）全年完成生产总值835亿元，增长4%；财政总收入119.9亿元，增长2.6%，其中公共财政预算收入84.5亿元，增长1.5%；规模以上工业增加值293亿元，增长3%；固定资产投资704亿元，增长5%；社会消费品零售总额170.3亿元，增长10.6%。

　　这一年，是改革创新扬帆起步的一年。一年来，我们未雨绸缪，破除思想藩篱，密集推动一系列改革举措，全市上下思改革、谋改革、促改革的氛围更加浓厚，各类市场主体发展活力竞相迸发。引进内资532亿元，增长21.6%；非公经济完成增加值占生产总值的比重达63.3%；新登记市场主体1.1万户，新登记资金125.1亿元，分别增长29.3%和56.5%，每百人拥有市场主体户数是全省平均水平的1.7倍。

　　这一年，是转型升级砥砺前行的一年。一年来，我们坚持转型发展、可持续发展不动摇，经济结构持续改善。战略性新兴产业占规模以上工业的比重达64.2%，提高4.1个百分点；第三产业增加值占生产总值的比重达36.9%，提高3.2个百分点；税收收入占财政总收入的比重77.5%，提高3.5个百分点。

　　这一年，是创优城市内涵提升的一年。一年来，我们以打造“精美特新”城市为目标，推动城市建设由外延扩张向内涵提升、功能完善转变，城市发展的集聚效应和承载能力不断增强。智慧城市建设成效初显。城区“断头路”基本打通，主要路口拓宽工程、主干道排涝点改造基本完成，新增公共停车泊位1900个。城镇化率达66.4%。

　　这一年，是情系民生福祉倍增的一年。一年来，我们坚持富民为先，顺应人民群众追求美好生活的期盼，让发展成果更多更公平惠及全市人民。全市各级财政民生事业支出83.2亿元，增长10%。省政府下达的76项民生任务全面完成；市政府确定的50件民生实事，除渝水五小和穗南城农贸市场建设因客观原因未完成外，其它均已完成。城镇居民人均可支配收入24762元，增长10.2%，农民人均纯收入11173元，增长11.2%；城乡居民储蓄存款余额339.6亿元，增长9.8%。城镇登记失业率控制在3.8%。居民消费价格涨幅控制在2.6%。

　　过去的一年，我们始终坚持打基础、谋长远，紧紧围绕调结构、促转型、抓改革、惠民生，扎实做了六方面工作。

　　（一）全力以赴稳增长调结构，三次产业协调发展。工业经济平稳发展。规模以上工业主营业务收入1460亿元，增长1.8%。突出抓好钢铁、新能源、新材料三大支柱产业帮扶工作，“一企一策”帮助重点困难企业解决用工、办证、融资等问题。新钢实现整体扭亏，赛维脱困取得阶段性成果，江锂、赣锋锂业等企业加快发展。沃格光电完成股份制改造，永盛矿冶、恩达麻世纪进入上市辅导备案阶段，6家中小微企业签订“新三板”挂牌协议。新钢成功发行30亿元短期融资券。在全省率先建成企业信用与金融服务一体化平台。中小企业公共服务平台投入运行。建成标准厂房57万平方米。新增入规企业35户。服务业加快发展。出台加快服务业发展意见，第三产业实现增加值308亿元，增长7%。出台扶持电子商务产业发展办法，实现交易额60.8亿元。兴邦信息列为国家级电子商务示范企业，金利达、菜小二列为省级示范企业；双林镇列为省级电子商务创业示范基地。在全省率先编制商业网点规划，中小商贸流通企业服务平台建设走在全国前列。腾达电器入选商务部首批“百城千店”示范工程。市物流协会成立，万商红商贸物流中心开业，赣西中心物流园区列为省级现代服务业集聚区。天工文化创意产业园被命名为省级文化产业示范基地，抱石文化创意园入驻文化企业7家。电视连续剧《仙女湖》在央视一套、八套黄金时段播出。新增全国休闲农业和乡村旅游示范点1个。接待游客增长25.8%，旅游总收入增长50.3%。新增小额贷款公司2家。各项融资余额1216亿元，增长5.3%。农业经济集约发展。粮食产量实现“十连增”。新增省级农业产业化龙头企业7家，45家省级以上龙头企业实现销售收入113亿元，带动18.2万农户增收4.3亿元。新增农民专业合作社432家、省级示范社19家。新增国家级园艺标准园1个。新增花卉苗木7.7万亩、高产油茶2.4万亩，“新余蜜桔”获国家地理标志证明商标。昌坊天香园蔬菜基地光伏大棚实现并网发电。农用土地流转21.7万亩，流转率达27%。

　　（二）全力以赴推项目扩投资，发展后劲不断增强。重点项目稳步推进。90个重点项目完成投资96.7亿元。蒙华铁路（新余段）完成线路定测，煤储基地项目纳入蒙华铁路一同立项审批。杭南长客运专线（新余段）主体工程基本完工。小型机场项目列入省航空运输布局规划。分电2台100万千瓦机组、赛维自备电厂项目已上报国家能源局。赣锋锂业万吨锂盐生产线、信达长林退城进区搬迁技改、渝水高科涂布纸等项目全部或部分投产。招商引资力度加大。签约引进项目161个，其中亿元以上项目79个，英泰能锂电池、普胜触摸屏、旭粤新能源节能玻璃等136个项目开工建设，新签约项目开工率达84.5%。资金政策争取成效明显。共争取上级资金46亿元，增长3.6%。“三项政策”累计获得国家补助资金20.3亿元，节能减排财政政策综合示范工作绩效考评全国第一。成功争取国家智慧城市试点、全国水生态文明城市建设试点，被国家发改委确定为新能源示范城市，分宜县、渝水区确认为原中央苏区县，高新区入选国家第一批分布式光伏发电示范区，仙女湖饮用水源保护区成功入选全国生态良好湖泊保护试点。争取省级用地指标4756亩。

　　（三）全力以赴抓建设提内涵，城乡统筹加速发展。城市功能不断完善。编制或调整中心城区控制性详细规划14个，编制完成城市风貌、综合交通、主城区排水等专项规划8个；市、县、乡三级土地利用总体规划修编全面完成。袁河生态新城基础设施基本建成。高铁站房、站前广场、钟灵大道主体工程基本完工。站前路填平工程、抱石大道东延、站前路西延等建成通车。改造维修城区主干道路面2.37万平方米。廖家江长青桥至五一桥段江体改造完成。新建雨污管网7公里。建成城区小公园3个。新增绿地面积3万平方米，主城区绿地率、绿化覆盖率、人均公园面积分别达52.46%、54.07%、18.95平方米。镇村联动稳步推进。乡镇总体规划修编全面完成，中心镇控制性详细规划编制基本完成。罗坊、双林两镇实行计划单列管理。11个中心镇共安排建设项目105个，完成投资10.1亿元。4个镇列为全省百强中心镇重点扶持。樟排线（罗坊至水西段）一级公路改造实现通车，新建农村公路210.4公里，维修县、乡道68.5公里。建设新农村点186个，重点打造精品示范村10个。新增农村自来水受益人口8.2万。九龙山乡、昌坊村列为全国“美丽乡村”创建试点单位。建有国家级生态乡镇4个、国家级生态村1个。农业设施日益完善。53座小（2）型病险水库和4座中型病险水闸除险加固、5条中小河流治理项目顺利推进。小农水重点县项目年度建设任务完成。 建设高标准农田4万亩，完成土地整理9.1万亩，新增耕地6238亩，治理水土流失3.6万亩。荣获全省耕地保护成效显著先进设区市。

　　 （四）全力以赴强科技引人才，创新能力稳步提高。科技进步对经济增长贡献率达53.5%，科技进步统计监测综合排名蝉联全省第二，省科技奖二等奖以上获奖数量列全省第一。创新平台不断筑牢。高新区国家级科技企业孵化器获批。国家光伏工程技术研究中心通过科技部评估验收。获批组建省级工程技术研究中心2家，组建市级中心4家。自主创新能力显著增强。国家级高新技术企业达19家，新增省重点新产品39项。获批国家级科技项目13个。每万人专利申请量和授权量均居全省第一。新认定江西省著名商标20个。2家企业列入国家首批知识产权贯标试点单位。人才工作成效明显。12名高端人才和4个战略性新兴产业团队入选“赣鄱英才555工程”，2人被评为省突出贡献人才。新增1人获国务院特殊津贴、3人获省政府特殊津贴，2名外国专家获“庐山友谊奖”。新增1家博士后工作站、1个国家级技能大师工作室和1个国家级高技能人才培训基地。

　　 （五）全力以赴惠民生促和谐，社会事业全面进步。就业创业力度加大。出台促进创新创业和加强用工服务两个政策文件，新建创业孵化基地和外来务工人员之家，列为全国百家城市技能振兴示范城市。新增城镇就业2.4万人，新转移农村劳动力1.6万人；新发放小额贷款4亿元，扶持和带动1.5万人就业创业。社会保障成效显著。在全省率先启动重特大疾病医疗救助工作，率先建立城乡医疗救助同步结算制度，率先开展社会保险“多险合一”试点，率先推行医疗保险付费方式改革，率先建立乡村医生养老保险制度，率先开展农村老年人颐养之家建设，率先实行居民生活用水阶梯水价，率先在中心镇和企业启动公共租赁住房建设。企业退休人员养老金待遇“九连提”。开工新建廉租住房3300套、公共租赁住房4550套，发放租赁补贴6568户、1132万元；改造棚户区1996户、15.29万平方米，农村危房改造1600户。新建或改造城区农贸市场10个。生态文明成果丰硕。大力实施净空、净水、净土“三净”行动，省下达的单位GDP能耗下降、四项主要污染物削减任务全面完成。生活垃圾发电等合同环境服务7个试点项目顺利推进，高新区污水处理厂技改、新钢5号和6号烧结机烟气脱硫设施建设等重点减排项目建成。社会事业协调发展。城乡义务教育均衡发展，北师大新余附属学校、渝水六小、特教学校新校区投入使用。职业教育现代学徒制试点和集团化办学取得良好成效。学前教育三年行动计划任务基本完成，少儿舞蹈获“群星奖”等多项全国最高奖项。县级公立医院改革有序推进，基本药物制度实现基层全覆盖并向县级公立医院延伸。深化治理医药购销领域商业贿赂，实现“阳光医药”网上监察。扶贫开发工作稳步推进。成功申报为国家公共文化服务体系示范区创建城市。文艺创作评比全省第一。第十二届全运会上获金牌2枚。人口计生工作全省领先。社会治理不断创新。社区网格化管理深入推进，推行民警、城管进社区，开展物业进社区试点。深入开展社会治安专项整治，全国“畅通工程”一等管理水平城市创建工作通过国家部委联合验收。探索治安、交通、医疗矛盾纠纷集中调解取得显著成效，国家安全人民防线、信访维稳、安全生产、食品药品安全监管进一步加强，没有发生影响稳定的群体性事件，全市社会政治稳定。

　　 （六）全力以赴促改革提效能，内生动力不断激发。推进行政审批制度改革，出台招商引资项目预审制、服务企业行政审批代理制，市级行政审批事项下放20%，取消行政审批项目18项。出台建设工程项目优化审批流程暂行方案，审批时间压缩至36个工作日。在全国率先建成覆盖市、县、乡三级数字化协同办公平台，实现行政审批网络化。清理机关事业单位临时用工783名。高新区“大部制设置、企业化管理”模式顺利推进。出台规范涉企检查等13个扶持企业发展文件。在全省率先开展公务用车制度改革。成立财政金融委员会，出台政府性债务管理办法，探索投融资平台“融、用、管、还”市场化运作模式。市本级一级预算单位“三公”经费全面公开，一般性支出压缩10%。“营改增”工作稳步推进。通过土地清理和政策规范，运作土地增加收入效果明显。整合工业、农业、科技等专项资金近1亿元集中调配使用。坚持依法行政，行政复议委员会试点成效明显。建立部门绩效考评社会评价机制。全面落实党风廉政建设责任制和八项规定，防治腐败工作水平不断提高。统计、审计、质监、海关、档案、检验检疫、民族宗教、外事侨务、涉台事务、驻外机构、机关事务、防灾减灾、地质找矿、妇女儿童、老龄、科普、气象、人防、证券保险、邮政通讯、烟草、盐业、海事、国防动员、双拥优抚、民兵预备役、社科研究、志愿服务、红十字会人道援助和慈善事业、残疾人等工作取得新成绩。

　　 各位代表，2013年，面对持续低迷的宏观经济环境，面对结构调整、转型升级的巨大压力，能够取得这样的成绩，实属来之不易。这是我们按照中央和省委、省政府的决策部署，在市委的坚强领导下，在市人大、市政协的监督支持下，在社会各界的大力帮助下，全市各级政府依靠广大人民群众，攻坚克难、积极作为的结果。在此，我代表市人民政府，向辛勤工作在全市各条战线上的广大干部群众，向人大代表、政协委员和离退休老同志，向各民主党派、工商联、无党派和社会各界人士，向中央和省驻市单位、来余投资企业，向驻市解放军、武警官兵和公安干警，表示衷心的感谢并致以崇高的敬意！

　　 在肯定成绩的同时，我们也清醒认识到，全市经济社会发展还面临不少困难和问题：产业结构不尽合理，新兴产业总量偏小，重大项目支撑乏力，转型升级任务艰巨；社会转型矛盾叠加，生态治理“欠账”较多，政府性债务重，社会保障水平有待提升，社会治理创新还有大量工作要做；少数干部改革创新意识不强、服务发展水平不高、破解难题能力不足等问题还不同程度存在。对此，我们一定认真对待，切实加以解决。

　　 二、2014年工作任务

　　 2014年，是全面贯彻落实党的十八届三中全会精神的开局之年，是全面深化各项改革的关键之年。政府工作的总体要求是：全面贯彻落实党的十八大、十八届三中全会、中央经济工作会议和省委、市委全会精神，牢牢把握稳中求进工作总基调，坚持“发展至上、富民为先”，围绕打造“全省改革创新先行先试示范区”，以改革创新为统领，以结构调整为抓手，以转型升级为主题，着力激发市场活力，着力改善民生，切实提高经济发展质量和效益，实现经济持续健康发展、社会和谐稳定。全市经济社会发展的主要预期目标是：生产总值增长10%；财政总收入增长15%，公共财政预算收入增长13%；规模以上工业增加值增长12%，主营业务收入增长13%；固定资产投资增长18%；社会消费品零售总额增长13.5%；外贸出口总额增长7.5%；实际利用外资增长10%，引进内资增长15%；城镇居民人均可支配收入增长10%以上，农民人均纯收入增长11%以上。居民消费价格指数、人口自然增长率控制在省定目标以内，完成省下达的节能减排计划任务。

　　 围绕上述目标，重点做好以下几方面工作：

　　 （一）突出改革创新，激发发展活力

　　 切实贯彻省政府《关于支持赣西经济转型加快发展的若干意见》和我市《关于进一步推进改革创新的意见》，制定相关实施方案，充分发挥市场在资源配置中的决定性作用，积极推进“小政府、大社会、活市场、优环境”等方面22项改革。

　　 深化行政体制改革。优化政府组织结构，三季度完成机构改革；完成仙女湖区、孔目江区、省职教园区行政资源整合。继续简政放权，自行设置的行政审批事项一律取消；国家和省里下放市里的行政审批事项，能下放县（区）的一律下放。工业园区全面推行“大部制设置、企业化管理”新机制。推进事业单位“去行政化”和人事制度、绩效工资制度改革。整合撤并驻外机构，支持组建异地商（协）会，强化产业协会自治自管，逐步将政府部门有关事务性、辅助性职能转移、授权或委托给商（协）会。完善绩效考核评价体系，加大民生改善、社会和谐、生态文明等指标权重。

　　 推进财税体制改革。全面调整和优化财政预算制度，扩大预算绩效管理试点范围，理顺市与县（区）事权财权，增强市本级财政调控能力。创新财政支持经济发展模式，盘活存量资金，整合专项资金，实现多部门归口管理向一个口子管理转变；除中央和省另有规定外，支持民营企业发展的财政资金，主要采取贷款贴息、担保、股权投资等方式安排。

　　 着力防范债务风险。坚持科学举债，严控新增规模，优化债务结构。发挥财政金融委员会作用，落实政府性债务管理办法，清理整合各类政府性投融资平台，试行投融资负面清单管理制度，健全风险防控预警体系，统筹债务管理。偿债计划纳入财政预算，完成情况纳入绩效考核。落实偿债准备金6亿元。

　　 创新社会治理体制。加快政社分开，大力发展各类社会组织。建立“县（区）—街道—社区”三级管理体系，推进社区网格化管理和信息化建设，实现社区服务从政务服务向公益、便民服务转变。培育发展志愿者组织。全面有序放开城区落户限制。

　　 健全现代市场体系。深化城市公用事业和公共服务领域改革，推进政府投融资项目工程预决算评审、城市公共园林绿化设施养护管理、新农合大病保险等三项政府购买服务试点。开展农村住宅和土地承包经营权确权登记工作。建立健全土地承包经营权流转交易市场和信息化平台，引导土地向新型农业经营主体流转，土地流转率达35%。加快工商登记、监管、服务制度改革。

　　 （二）突出转型升级，发展新型工业

　　 坚持主攻工业不动摇，大力发展实体经济，着力调优工业结构，全面提升工业经济可持续发展水平。

　　 推动工业转型升级。积极推动钢铁、新能源、新材料三大产业向终端深加工延伸，提升产品附加值。大力发展光电信息、高端装备制造、节能环保等战略性新兴产业，坚决淘汰落后产能，将有限的资源配置到新兴产业上。应用高新技术改造提升钢铁、电力、纺织等传统产业，提高现代化水平。大力发展工业设计，举办工业设计创新大赛。坚持扶优扶强，推动各类要素向优强企业、优秀企业家集聚，培育一批优强企业集群。加快中小微企业孵化基地建设，发挥中小企业公共服务平台功能，推动中小微企业蓬勃发展，新增入规企业50户。组织实施沃格光电玻璃精加工、高志环保电镀工业园等重点工业项目40个，完成投资32亿元。加快沃格光电、永盛矿冶、恩达麻世纪等企业上市步伐，推动1家以上企业在“新三板”成功挂牌。

　　 激发园区发展活力。完善园区规划体系，加强基础设施建设，出台支持“飞地经济”发展政策，推动新上工业项目向园区集中。着力盘活闲置土地和厂房，提高资源利用效率。抓好标准厂房建设，确保标准厂房面积达100万平方米。高新区重点发展新能源、钢铁和装备制造、新材料和化工、光电信息、生物医药食品、现代服务业等六大主导产业；分宜工业园重点围绕构建“一园三区”发展新格局，推进扩区调区工作，打造特色产业园区；袁河经济开发区全面完成机械加工园、机电产业园一期建设。三大园区分别实现主营业务收入600亿元、240亿元和180亿元。

　　 全力帮扶企业发展。创新帮扶理念，将更多事项交由市场调节。建立“五个一”帮扶工作机制，着力破解制约企业发展的人才、资金、产业配套等瓶颈问题。强化校企对接，加强异地劳务合作，千方百计满足企业用工需求。积极推进新钢社会管理职能移交，推动赛维全面恢复生产和股权结构优化，推动江锂、华电等企业引入战略合作伙伴和推广产品应用。支持光伏企业投资西部地区光伏电站建设。鼓励企业建立产业联盟，推动企业协同发展、良性发展。高度关注困难企业资金链安全。

　　 （三）突出结构优化，壮大第三产业

　　 把发展服务业作为打造经济“升级版”的战略举措，以优化布局、提升水平、扩大总量为目标，完善“1＋9”服务业发展政策体系，不断提升服务业在全市经济中的支撑作用。

　　 加速发展生产性服务业。加快金融机构引进步伐，设立1-2家村镇银行。建立担保风险代偿和补贴机制，支持市担保中心做大做强。加强政银企协作，重视外汇融资服务功能，发挥企业信用与金融服务一体化平台作用，推动金融资本更多投向实体经济。鼓励社会力量发起或参与建立产业发展基金、风险投资基金、私募股权投资基金、创业投资公司等。年末实现各项融资余额1320亿元，其中贷款余额900亿元，增长12.5%。以赣西中心物流园区、高新区物流中心为载体，加大龙头物流企业引进培育力度。推进物流综合信息平台、陆路口岸作业区建设，扩大铁海联运规模。加快发展快递物流业，引导品牌企业在余设立赣西区域快件处理中心。

　　 提升发展消费性服务业。严格落实商业网点规划，适度控制交易市场建设，积极引进新型商业业态，促进商贸服务业转型发展。深入推进“1020工程”，完成投资8亿元和实现销售额160亿元。着力抓好万商红商贸物流中心、正大钢材市场、赣西商贸城等项目的培育发展。推进农产品现代流通综合试点、城区农贸市场建设改造，完善中小商贸流通企业服务平台建设。制定旅游产业三年行动纲要。加强旅游基础设施建设和宣传推介，抓好仙女湖5A级景区创建。大力发展文化旅游、生态旅游、农业休闲旅游等新兴业态，加快开发夏布艺术品、陶瓷艺术品等特色旅游商品，提升旅游产业对经济发展的贡献度。实现接待游客增长20%、旅游收入增长25%以上。

　　 创新发展新兴服务业。实施电子商务全民创业工程，支持各类市场主体发展电子商务。推进中国国际电商城建设，加大居无忧商城“网上经济特区”和电商实体企业招商力度，推动中国（新余）家居总部基地、金利达电子商务产业园、双林镇等电子商务基地建设，推动万商红、腾达电器等企业打造电子商务交易平台。实现电子商务交易额100亿元以上。加快发展八大重点文化产业，大力引进文化产业领军人才，培育10家文化产业领军企业。办好中国苎麻夏布文化节、第三届傅抱石文化艺术节、第二届工艺美术作品大赛。推进仙女湖国际汽车文化产业园建设。

　　 （四）突出统筹发展，推进城乡一体

　　 围绕在全省率先实现城乡一体化目标，着力破解城乡二元结构，加快形成以工促农、以城带乡、工农互惠、城乡一体的新型工农城乡关系。

　　 提升城市发展内涵。以创建全国文明城市和打造“精美特新”城市为目标，走特色化、内涵式发展道路，控制城区边界、完善基础功能、注重发展效益，城镇化率达67.9%。理顺城乡规划管理体制，强化城乡规划“一张图”。启动城市总体规划修编，基本完成老城区控规修编，完成城区排水防涝综合规划、主干道景观规划和市政基础设施、消防等专项规划编制。计划安排城建项目39个，年度投资14.5亿元。加大袁河生态新城宣传推介和招商引资力度，吸引更多市场主体投资建设。在杭南长客运专线开通前完成高铁新区基础性配套项目建设。规划建设过境重载车道，推进环城西路北延跨铁路立交桥、滨江路西延段、阳光大道西延段等项目建设，确保余新公路建成通车。加强城市配电网建设。推进新钢铁山、路东棚户区改造。深入推进智慧新余建设，充分发挥“数字城管”监督联动作用，进一步提升城市管理水平。加强和规范物业管理，推进物业服务纳入社区统一管理。

　　 加快镇村联动步伐。坚持产业发展和镇村建设相互融合理念，强化镇村特色产业培育，加快形成“一乡一业，一村一品”发展格局。全面实施乡村建设规划许可管理，基本完成村镇规划编制。抓好农民新社区和集中供水、污水处理、卫生院所等公共服务设施建设。健全多元化融资体系，吸纳社会资金参与中心镇建设。重点扶持罗坊、双林两个示范镇建设和发展。扎实推进新农村建设，重点打造18个中心示范村。以垃圾、污水、粉尘治理为重点，推进农村环境整治，保护好清洁水源、清洁家园、清洁田园。坚持产业扶贫，抓好贫困村帮扶工作。

　　 大力发展现代农业。深入实施农业产业化“1010”工程，新增省级以上龙头企业4家。完善规划定位，发展规模经营，加快五大现代农业园发展。以基地建设为抓手，重点发展七大特色农业产业。全力推进光伏生态农业示范工程建设。加大新型农民培养力度，培育一批新型职业农民和农业合作经济组织领头人。着力培育新型农业经营主体，大力发展家庭农场，新增农民专业合作社150家、休闲农家乐30家。抓好43座小（2）型病险水库、4条中小河流治理和小农水重点县等项目建设。

　　 （五）突出项目建设，增强发展后劲

　　 坚定不移实施项目带动战略，形成富有生机活力的项目建设机制，推进生产要素集聚、投资增长、产业升级。

　　 加强重点项目建设。坚持重点项目领导挂点制、项目建设分级管理制、政府投融资项目代建制。全面落实招商引资项目预审制和服务企业行政审批代理制，实施重大项目联审、限时办结等制度，完善跟踪服务，强化督查考核，全力推动项目建设。计划安排投资1.5亿元以上项目62个，年度计划投资73.3亿元。重点项目分批次下达，首次计划安排52个，年度计划投资40亿元，其中新建项目26个。倾力抓好事关新余长远发展的重大项目，保障杭南长客运专线（新余段）投入运营，做好蒙华铁路（新余段）、分电2台100万千瓦机组、赛维自备电厂开工保障，抓好大唐新余2台100万千瓦煤电一体化、煤储基地及相关配套项目等前期工作。

　　 提高招商引资实效。推进招商引资由政府主导向市场驱动转变，完善经济激励政策，推行委托商（协）会招商、聘请产业顾问招商。严格落实招商引资“一把手”负责制，组建以商务部门为主体的专业招商队伍，强化对县（区）、园区等招商主体的考核。充分依托各项国家政策、三大产业、工业地产和闲置土地厂房等资源招商，重点抓好光电信息、节能环保、机械装备、电子商务、文化创意、艺术陶瓷等产业招商。加强与国药集团、国机重工、中核新能源等央企对接，深化医药、机械、新能源等领域合作。

　　 积极争取项目资金。吃透上级政策，全力做好项目储备和前期工作，力争获得更多项目、资金和建设用地指标支持，确保争取上级资金增长10%。完善考核调度机制，确保“城市矿产”示范基地、资源枯竭城市转型试点顺利通过中期评估验收。力争小型机场项目列入国家规划。

　　 （六）突出科技引领，提升创新能力

　　 实施科技协同创新“六大引领工程”，加快建立健全鼓励原始创新、集成创新、引进消化吸收再创新的体制机制，促进科技和经济紧密结合。

　　 强化科技协同创新。整合市、县（区）财政科技资金，统一集中调配使用。强化企业创新主体地位，发挥省级以上研发平台创新驱动能力，新增省级研发平台1个、市级研发平台5个。依托新钢、赛维、江锂、亚林中心、新余学院等单位组建一批协同创新团队。加快建设知识产权维权中心，开展企业知识产权试点示范和托管，实施重大专利产业化项目，培育一批知识产权优强企业。申请专利800件。

　　 推进产学研深度融合。加大科技项目申报力度，力争获批国家级项目8项以上、省级项目50项以上。探索建立主要由市场决定技术创新项目和经费分配、成果评价的机制。新增国家级高新技术企业5家，组建省级生产力促进中心5家。发展技术市场，健全技术转移机制，完善风险投资机制，促进科技成果资本化、产业化。深化与高校、科研机构的合作，力争20项以上科技成果在我市孵化。

　　 加强人才队伍建设。完善人才选拔、评价机制，深化人才管理改革试验区建设，全面落实人才引进培养政策和人才发展规划。推进技能振兴试点工作，实施高技能人才振兴工程和紧缺技能人才培养计划，加强技能大师工作室、高技能人才培养示范基地建设。加快职业经理人队伍建设，重点培育一批具有现代经营理念和战略眼光的经济发展领军人才和优秀企业家。

　　 （七）突出生态文明，建设美丽新余

　　 以推进全国水生态文明城市建设试点和合同环境服务试点为契机，加快生态文明制度体系建设，推动形成人与自然和谐发展现代化建设新格局。

　　 深化节能减排。严格执行节能减排目标责任制，严厉监管并及时通报企业污染物排放和防治设施运行情况，督导重点企业落实节能减排目标。加快重点减排项目建设，确保新钢4号烧结机脱硫设施、分电9号机组新增炉外烟气脱硫设施、海螺2号生产线低氮燃烧改造及烟气脱硫设施建成运行。抓好城西污水处理厂及配套管网建设。

　　 发展低碳经济。严格环境准入、考核问责制度，加强生态空间管控，大力发展循环经济。全力抓好“三项政策”项目建设，推动节能减排产业发展壮大。抓好生活垃圾发电等7个合同环境项目建设，推进环境污染第三方治理。加快太阳能建筑规模化应用，完成全国第一批分布式光伏示范区72兆瓦电站建设，实施光热应用建筑面积150万平方米。推进养殖业生态化改造。加大新能源重型货车、出租车和公交车投运力度。

　　 加强生态保护。坚持保护为先，制定“五山”保护利用综合规划和相关制度，推动生态治理和修复。深入推进“森林城乡、绿色通道”建设，完成造林面积6万亩，实施通道绿化50公里、提升改造80公里，建成仙女湖大道西延段高标准绿道15公里；打造100个“森林十创”点。确保仙女湖、孔目江两个主要饮用水源保护区水质稳定在Ⅱ类以上。严格实施高污染排放机动车区域限行措施，加速淘汰黄标车。完善空气质量监测体系。加强农村面源污染治理。

　　 （八）突出民生改善，发展社会事业

　　 把人民生活质量提升、幸福感不断增强作为经济发展的最终目的，多谋民生之利，多解民生之忧，让群众更幸福、城市更包容、社会更温暖。

　　 办好民生实事。围绕解决好人民群众最关心最直接最现实的利益问题，突出扩大就业和促进创业、完善社会保障体系、强化医药卫生保障、促进教育公平和均衡发展、发展公益文化、加快保障性住房建设、改善居民出行条件、改善群众生产生活条件等方面，办好60件实事，实现发展成果更多更公平惠及全市人民。

　　 发展社会事业。大力促进教育公平，统筹城乡义务教育资源均衡配置，逐步缩小区域、城乡、校际差距。完善城区中小学教育网点布局，推进城区中小学改、扩建，缓解适龄儿童入学压力和中小学“大班额”现象。整合职业教育资源，推动职业教育向企业化、集约化、特色化发展。创新高校人才培养机制，促进新余学院办出特色。推进继续教育改革发展。加快文化体制改革，开展国家公共文化服务体系示范区创建，全力打造“数字文化网”。开展市级公立医院改革，推进药品采购、管理和新农合付费方式等改革，加强卫生服务能力水平建设。打造全省领先的食品药品“智慧监管”工程，确保广大人民群众“舌尖上的安全”。完善“招工、培训、就业”三位一体模式，构建劳动者终身职业培训体系。完善以居家为基础、社会为依托、机构为支撑的养老服务体系。落实好一方是独生子女的夫妇可生育两个孩子的政策。力争在省第十四届运动会上实现金牌总数排名“保四争三”目标。争创全国“双拥”模范城市七连冠。抓好精神文明建设，提高市民素质。关心支持妇儿、老龄事业，发展残疾人事业和慈善事业。加强国防后备力量和人民防空建设。抓好全国第三次经济普查。做好新形势下的科普、气象、档案、史志、外事侨务、民族宗教、防灾减灾、邮政通讯等工作。

　　 确保社会稳定。深入推进社会管理创新和平安新余、法治新余建设，维护社会公平正义。改进社会治理方式，完善“源头治理、动态管理、应急处置”三道防线。加强基层社会服务管理体系建设，提升管理水平和公共服务能力。加强社会治安综合治理，抓好以“智慧天网工程”为引领的城市防控体系建设，依法严密防范和惩治各类违法犯罪活动。继续加强道路“畅通工程”建设，进一步提高群众安全出行、文明出行意识。深化安全生产管理体制改革，建立隐患排查治理体系和安全预防控制体系，促进安全生产形势持续稳定好转。完善信访制度，健全矛盾纠纷调解工作体系，畅通和规范群众诉求表达、利益协调、权益保障渠道。深入开展“六五”普法教育，不断完善司法救助制度。

　　三、加强政府自身建设

　　 以开展党的群众路线教育实践活动为契机，切实转变机关作风，提高施政水平和行政效率，建设职能科学、结构优化、廉洁高效、人民满意的服务型政府。

　　 推进依法行政。健全行政决策程序，完善重大决策听证、社会稳定风险评估机制，推进行政决策科学化、程序化、制度化。认真贯彻落实市委决策部署，积极发挥人大、政协民主作用，高质量办理人大议案、政协提案及其意见建议。自觉接受人大、政协监督，主动接受社会公众和新闻舆论监督，广开渠道听取群众意见建议。完善行政执法程序，规范执法自由裁量权，加强行政执法监督，全面落实行政执法责任制和执法经费由财政保障制度，做到严格规范公正文明执法。

　　 提升政府效能。全面落实岗位责任制、首问责任制、服务承诺制等制度，及时解决涉及企业发展、群众利益的急难事项。进一步优化行政审批流程，实现审批格式标准化、信息公开化。依托数字化协同办公平台，开发建设高度集成、部门联网、实时监察的网上审批系统、便民服务系统。加强督查督办，从严整治不作为、乱作为，确保政令畅通。

　　 加强廉政建设。健全完善惩治和预防腐败体系，加强建设工程招投标、财政转移支付等公共资源管理权力的监管，将权力关进制度的笼子里。建立健全土地、矿产资源节约集约利用新机制，健全国有资产管理体制、业绩考核评价机制和责任追究制。严格落实八项规定，狠刹形式主义、官僚主义、享乐主义和奢靡之风，努力实现干部清正、政府清廉、政治清明。建立刚性财政预算体系，增加民生投入，一般性财政支出一律压缩10%。推进市本级“三公”经费公开，做好决算公开准备工作。

　　 各位代表，做好今年的工作，责任重大、任务艰巨。让我们紧密团结在以习近平同志为总书记的党中央周围，在省委、省政府和市委的坚强领导下，解放思想，锐意改革，凝心聚力，攻坚克难，为加快建设和谐富裕文明新余、提前全面建成小康社会而努力奋斗！
